# Development and validation of MRI-derived deep learning score for non-invasive prediction of PD-L1 expression and prognostic stratification in head and neck squamous cell carcinoma

**DOI:** 10.1186/s40644-025-00837-5

**Published:** 2025-02-16

**Authors:** Cong Ding, Yue Kang, Fan Bai, Genji Bai, Junfang Xian

**Affiliations:** 1 Department of Radiology, Beijing Tongren Hospital, Capital Medical University, Beijing, 100730 China; 2 Department of Radiology, The Affiliated Huaian Hospital of Nanjing Medical University, Nanjing Medical University, No. 1 West Huanghe Road, Huaian, 223300 China

**Keywords:** Magnetic resonance imaging, Deep learning, PD-L1 expression, Immunotherapy, Head and neck squamous cell carcinoma

## Abstract

**Background:**

Immunotherapy has revolutionized the treatment landscape for head and neck squamous cell carcinoma (HNSCC) and PD-L1 combined positivity score (CPS) scoring is recommended as a biomarker for immunotherapy. Therefore, this study aimed to develop an MRI-based deep learning score (DLS) to non-invasively assess PD-L1 expression status in HNSCC patients and evaluate its potential effeciency in predicting prognostic stratification following treatment with immune checkpoint inhibitors (ICI).

**Methods:**

In this study, we collected data from four patient cohorts comprising a total of 610 HNSCC patients from two separate institutions. We developed deep learning models based on the ResNet-101 convolutional neural network to analyze three MRI sequences (T1WI, T2WI, and contrast-enhanced T1WI). Tumor regions were manually segmented, and features extracted from different MRI sequences were fused using a transformer-based model incorporating attention mechanisms. The model’s performance in predicting PD-L1 expression was evaluated using the area under the curve (AUC), sensitivity, specificity, and calibration metrics. Survival analyses were conducted using Kaplan-Meier survival curves and log-rank tests to evaluate the prognostic significance of the DLS.

**Results:**

The DLS demonstrated high predictive accuracy for PD-L1 expression, achieving an AUC of 0.981, 0.860 and 0.803 in the training, internal and external validation cohort. Patients with higher DLS scores demonstrated significantly improved progression-free survival (PFS) in both the internal validation cohort (hazard ratio: 0.491; 95% CI, 0.270–0.892; *P* = 0.005) and the external validation cohort (hazard ratio: 0.617; 95% CI, 0.391–0.973; *P* = 0.040). In the ICI-treated cohort, the DLS achieved an AUC of 0.739 for predicting durable clinical benefit (DCB).

**Conclusions:**

The proposed DLS offered a non-invasive and accurate approach for assessing PD-L1 expression in patients with HNSCC and effectively stratified HNSCC patients to benefit from immunotherapy based on PFS.

**Supplementary Information:**

The online version contains supplementary material available at 10.1186/s40644-025-00837-5.

## Background

Head and neck squamous cell carcinoma (HNSCC) ranks as the sixth most common cancer worldwide, with approximately 900,000 new cases and 500,000 deaths annually [1, 2]. Moreover, most patients with HNSCC are diagnosed at advanced stages [3]. Traditional treatments, including surgery, radiation, and chemotherapy, generally exhibit limited efficacy and are often accompanied by severe toxic side effects [4]. Consequently, the treatment landscape for HNSCC has been revolutionized by immunotherapy in recent years [5]. Pembrolizumab is recommended as a category IA treatment for combined positivity score (CPS)-positive HNSCC patients by both the National Comprehensive Cancer Network (NCCN) and the European Society for Medical Oncology (ESMO) guidelines [6]. However, only 20–30% of patients receiving immune checkpoint inhibitors (ICIs) derive clinical benefit [7, 8]. According to the American Society of Clinical Oncology (ASCO) guidelines, PD-L1 CPS scoring is recommended as a biomarker for immunotherapy in HNSCC patients [9]. Therefore, accurate assessment of PD-L1 expression status before treatment is essential for guiding personalized treatment plans.

Accurately predicting PD-L1 expression prior to treatment continues to pose challenges. Currently, PD-L1 expression is predominantly assessed via immunohistochemistry (IHC), which necessitates surgical or biopsy procedures [10]. This invasive method is not only time-consuming but also challenging for dynamic assessments [11]. Moreover, the reliability of this method is hindered by tumor heterogeneity, variability in antibody staining, and subjective result interpretation, all of which add to diagnostic complexity and uncertainty [12]. Given these limitations, there is a pressing need for non-invasive, reliable biomarkers to facilitate the effective selection of patients who may benefit from immunotherapy.

Recently, advancements in computer vision technology have enabled precise assessments of histological biomarkers using standard clinical imaging techniques, such as CT and MRI [13]. Previous studies have utilized tumor-based quantitative radiomics to extract features from CT images to predict PD-L1 expression status, achieving AUC values of 0.834 and 0.807 in the validation sets [14, 15]. Radiomics involves extracting handcrafted image features from tumors and selecting key features to train machine learning models. However, handcrafted radiomic methods require time-consuming tumor boundary delineation and only detect generalized features, which may lack reproducibility and repeatability [16, 17]. Deep learning integrates feature extraction and model construction within a unified convolutional neural network framework, allowing the automatic learning of more effective tumor image features by modifying the network architecture [18, 19]. Although previous studies have predicted PD-L1 status, their ability to forecast immunotherapy efficacy and provide prognostic stratification based on PD-L1 expression remains unconfirmed.

In this study, a deep learning score (DLS) based on MRI was developed to non-invasively assess PD-L1 expression status. Furthermore, the utility of the DLS in predicting progression-free survival (PFS) and durable clinical benefit (DCB) in patients treated with immune checkpoint inhibitors was investigated, aiming to provide more precise guidance for clinical decision-making.

## Materials and methods

### Study population

**Fig. 1:**
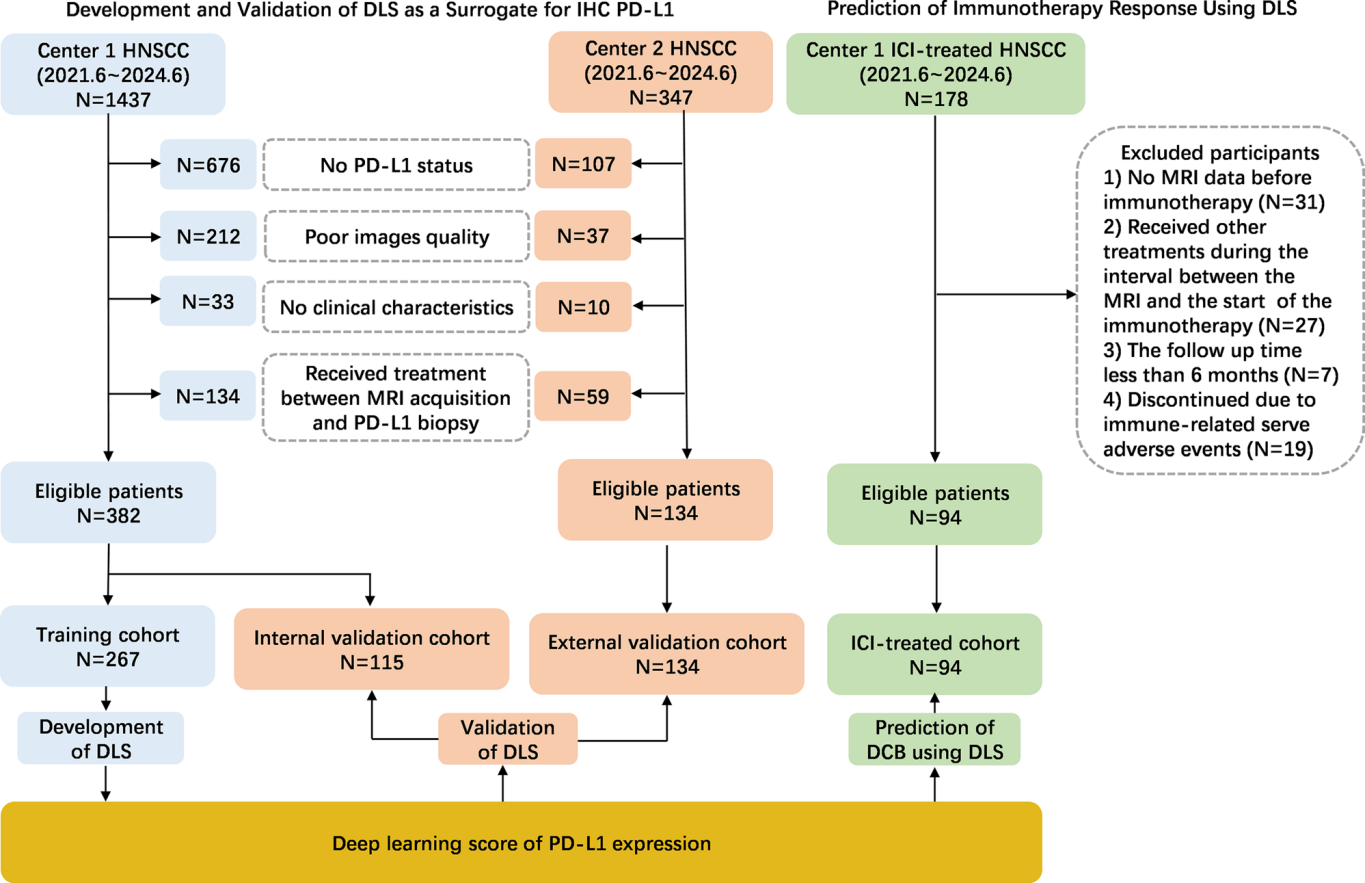
Study design and inclusion and exclusion diagram

This retrospective study received approval from the institutional review board (NCT06100497), and the requirement for written informed consent was waived. Four patient cohorts were collected from two institutions for this two-center retrospective study. Detailed inclusion and exclusion criteria are provided in Supplemental Methods S1. Among these, patients from institution 1 were divided randomly into training (*N* = 267) and internal validation (*N* = 115) cohorts at a 7:3 ratio, and the external validation cohort was composed of 134 patients from institution 2. Additionally, an ICI-treated retrospective cohort (*N* = 94) from institution 1 was used to evaluate the utility of the DLS in predicting DCB (Fig. 1).

Progression criteria for the ICI-treated cohort used to investigate the correlation between the DLS and durable clinical benefit (defined as PFS > 6 months) were based on the Response Evaluation Criteria in Solid Tumors (RECIST v1.1) [20]. PFS was defined as the duration from treatment initiation to the occurrence of disease progression, which included tumor growth, metastasis of the primary tumor, emergence of a new lesion, or patient death.

### PD-L1 detection and classification of expression

An experienced pathologist, blinded to imaging results and clinical data, analyzed histopathologic samples obtained from pretherapeutic biopsies of the primary tumor. During biopsy sampling, care was taken to avoid inflammation and ulceration on the surface of the tumor, and multiple biopsies were performed to ensure adequate tumor tissue for analysis. PD-L1 expression was retrospectively assessed using the VENTANA PD-L1 SP263 IHC assay, which is approved by the US Food and Drug Administration for the assessment of PD-L1 expression [21].

The PD-L1 combined positive score (CPS) was calculated using the formula:

CPS = [(number of PD−L1 positive staining tumor cells + PD−L1 positive staining tumor−associated immune cells) / total tumor cells] × 100.

All calculations were performed at a magnification of 40-fold. PD-L1 high-expression status was defined as a CPS of 20 or higher [22].

### Image data acquisition

The imaging protocol included axial fast spin-echo T1-weighted (T1WI), T2-weighted (T2WI), and fat-saturated contrast-enhanced T1-weighted (CE-T1WI) sequences. The CE-T1WI images were captured after administering a 0.1 mL/kg intravenous bolus of gadopentetate dimeglumine. Detailed acquisition parameters are available in Supplementary Table S2.

### Image segmentation and preprocessing

Tumor regions of interest (ROIs) were manually delineated slice-by-slice on contrast-enhanced T1-weighted images (CE-T1WI) using ITK-SNAP software (v3.8.0). Radiologists A and B with 3 and 5 years of experience in head and neck MRI, respectively, conducted the segmentations. These ROIs were then registered to T1-weighted and T2-weighted images using the same software. Any discrepancies were resolved by senior Radiologist C, who has 25 years of experience in this field. All radiologists were blinded to clinical and histopathological data. The segmented images were aligned with their respective T1-weighted and T2-weighted images, resampled to a voxel size of 1 × 1 × 1 mm³ using B-spline interpolation, and normalized on a 0–1 scale via min-max normalization.

### DL model construction and DL feature extraction

**Fig. 2:**
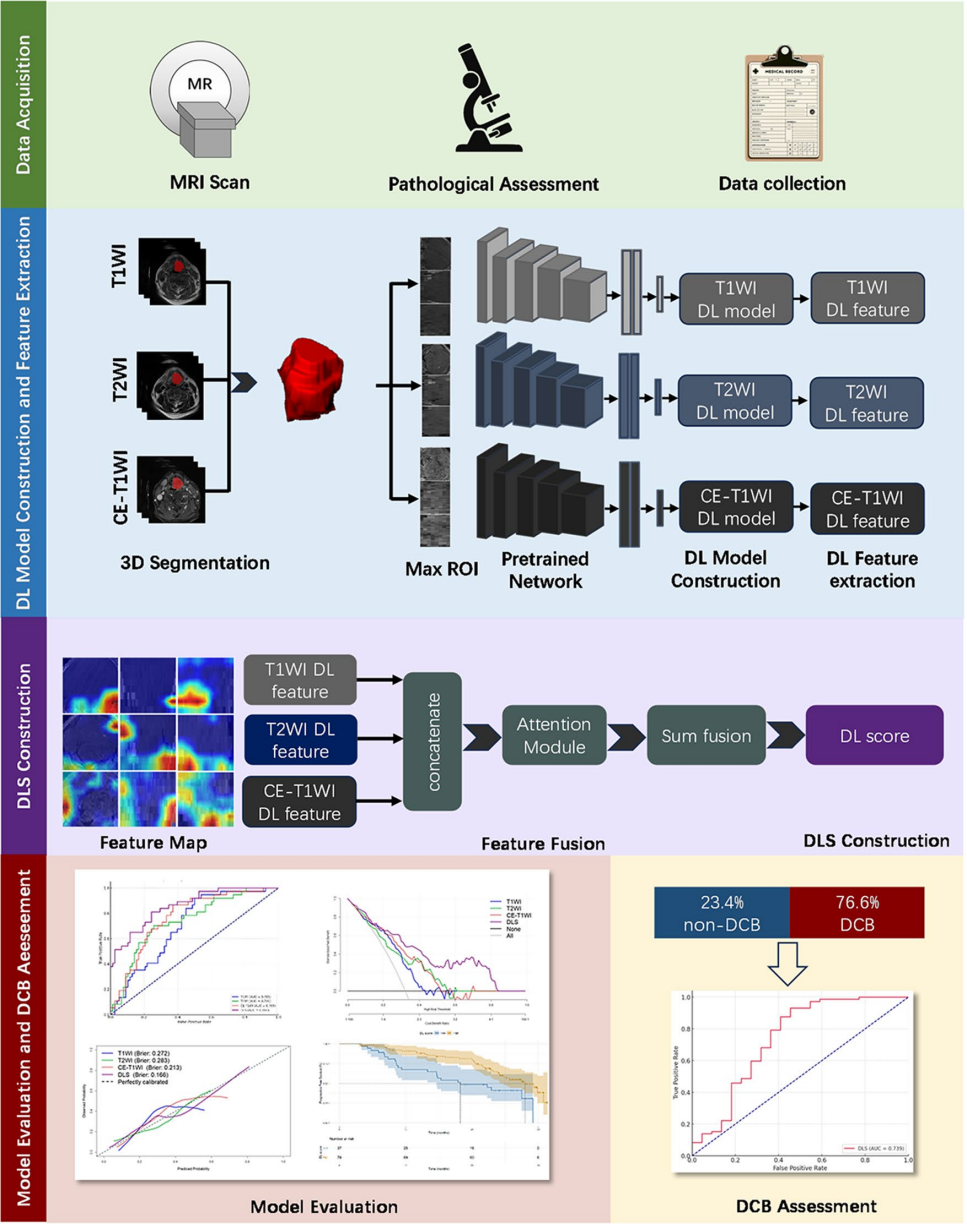
The schematic workflow of model development and validation

The study design is illustrated in Fig. 2. The ResNet-101 convolutional neural network was adopted as the primary architecture for the deep learning model. To enhance training effectiveness with limited data, transfer learning techniques were employed. The models were pre-trained on the ImageNet dataset to acquire initial weight values. This study utilized maximum orthogonal slices—axial, sagittal, and coronal planes with the largest tumor area—as 2.5D inputs for modeling. Before training, all inputs were resized to 224 × 224 pixels and underwent z-score normalization to ensure pixel value consistency. Additionally, we applied real-time data augmentation techniques, including random horizontal flipping and cropping. A focal loss function was utilized to address issues of class imbalance.

To enhance the interpretability of our model, Gradient-weighted Class Activation Mapping (Grad-CAM) was employed for visualization purposes. The class activation maps were produced by using gradient information from the final convolutional layer of the Convolutional Neural Networks (CNN) [23]. Additionally, the DL features extracted from these layers were then reduced to 128 dimensions using Principal Component Analysis (PCA), mitigating overfitting risks and boosting the model’s generalizability.

### Feature fusion and DLS construction

To integrate features from multiple MRI sequences, a transformer-based model was developed comprising eight attention heads and three encoder layers. Features from T1WI, T2WI, and CE-T1WI sequences, extracted using ResNet-101, were concatenated along the channel dimension. The concatenated features were segmented into fixed-size patches, with a multi-head self-attention mechanism applied to enhance representation by focusing on interdependencies and positional information. The refined feature maps were subjected to pooling operations and decoded by a multilayer perceptron (MLP) that outputs the DLS via a softmax function. Model parameters were updated using the Stochastic Gradient Descent (SGD) optimizer with an initial learning rate of 0.01 and a batch size of 32. To prevent overfitting, an early stopping strategy and Dropout technology were employed. The entire model was implemented in the PyTorch framework and trained efficiently on a system equipped with an NVIDIA GeForce RTX 4080 GPU.

### Statistical analysis

All statistical analyses and graphical outputs were generated using SPSS (version 25), R (version 4.1.2), and Python (version 3.8.5). Continuous variables between training and validation groups were analyzed using the Mann-Whitney U test or Student’s t-test, while categorical variables were assessed with the chi-square test or Fisher’s exact test as appropriate. Model performance was evaluated by calculating the AUC with a 95% confidence interval (CI), along with sensitivity, specificity, positive predictive value (PPV), negative predictive value (NPV), and accuracy. The DeLong test was employed for comparative analysis of AUCs. Probabilistic prediction accuracy was gauged using the Brier score (range from zero to one, with lower scores indicating better calibration) and calibration curves. The performance of DLS to predict DCB is evaluated by calculating the AUC. According to the Youden index from the training cohort, the patients were categorized into high DLS and low DLS groups. The Kaplan-Meier method and log-rank tests were employed to conduct survival analyses and compare PFS among patient groups stratified by the DLS. Statistical significance was established at a two-tailed *P*-value of less than 0.05.

## Results

### Patients characteristics

Table 1 presents the clinical characteristics of patients used for training and validating non-invasive PD-L1 status measurements. PD-L1 high expression prevalence, as determined by IHC in the training, internal validation, and external validation cohorts, was 32.2%, 32.2%, and 48.5%. Supplementary Table S2 details the clinical characteristics of patients assessed for the clinical utility of the DLS. The retrospective cohort1, treated with ICIs, comprised 94 patients, of whom 23.4% experienced a DCB.

**Table 1:** Clinical and pathologic characteristics of patients with HNSCC in the training, Internal validation, and external validation cohorts

Clinical Characteristics		Training Cohort (*n* = 267)	Internal validation cohort (*n* = 115)	External validation cohort (*n* = 134)		*P* value
Age (mean ± SD, years)		61.70 ± 8.50	59.85 ± 8.16	58.76 ± 8.63		0.003
Histological differentiation						0.505
	Well	31 (11.61%)	11 (9.57%)	14 (10.45%)		
	Moderately	115 (43.07%)	48 (41.74%)	47 (35.07%)		
	Poorly	121(45.32%)	56 (48.69%)	73 (54.48%)		
T stage						0.043
	2	89 (33.33%)	30 (26.09%)	46 (34.33%)		
	3	120 (44.94%)	46 (40.00%)	46 (34.33%)		
	4	58 (21.73%)	39 (33.91%)	42 (31.34%)		
N stage						0.321
	0	85 (31.84%)	34 (29.57%)	33 (24.63%)		
	1	83 (31.09%)	31 (26.96%)	40 (29.85%)		
	2	92 (34.46%)	42 (36.52%)	53 (39.55%)		
	3	7 (2.61%)	8 (6.95%)	8 (5.97%)		
Smoking						0.519
	No	72 (27.00%)	29 (25.22%)	42 (31.34%)		
	Yes	195 (73.00%)	86 (74.78%)	92 (68.66%)		
Drink						0.758
	No	185(85.5%)	79 (68.70%)	88 (65.67%)		
	Yes	82 (14.5%)	36 (31.30%)	46 (34.33%)		
Sex						0.196
	Male	238 (89.14%)	109 (76.20%)	123 (91.79%)		
	Female	29(10.86%)	6 (23.80%)	11 (8.21%)		

*P* value < 0.05 is considered as a significant difference.SD, standard deviation

### Deep learning model construction, comparison, and evaluation

**Fig. 3:**
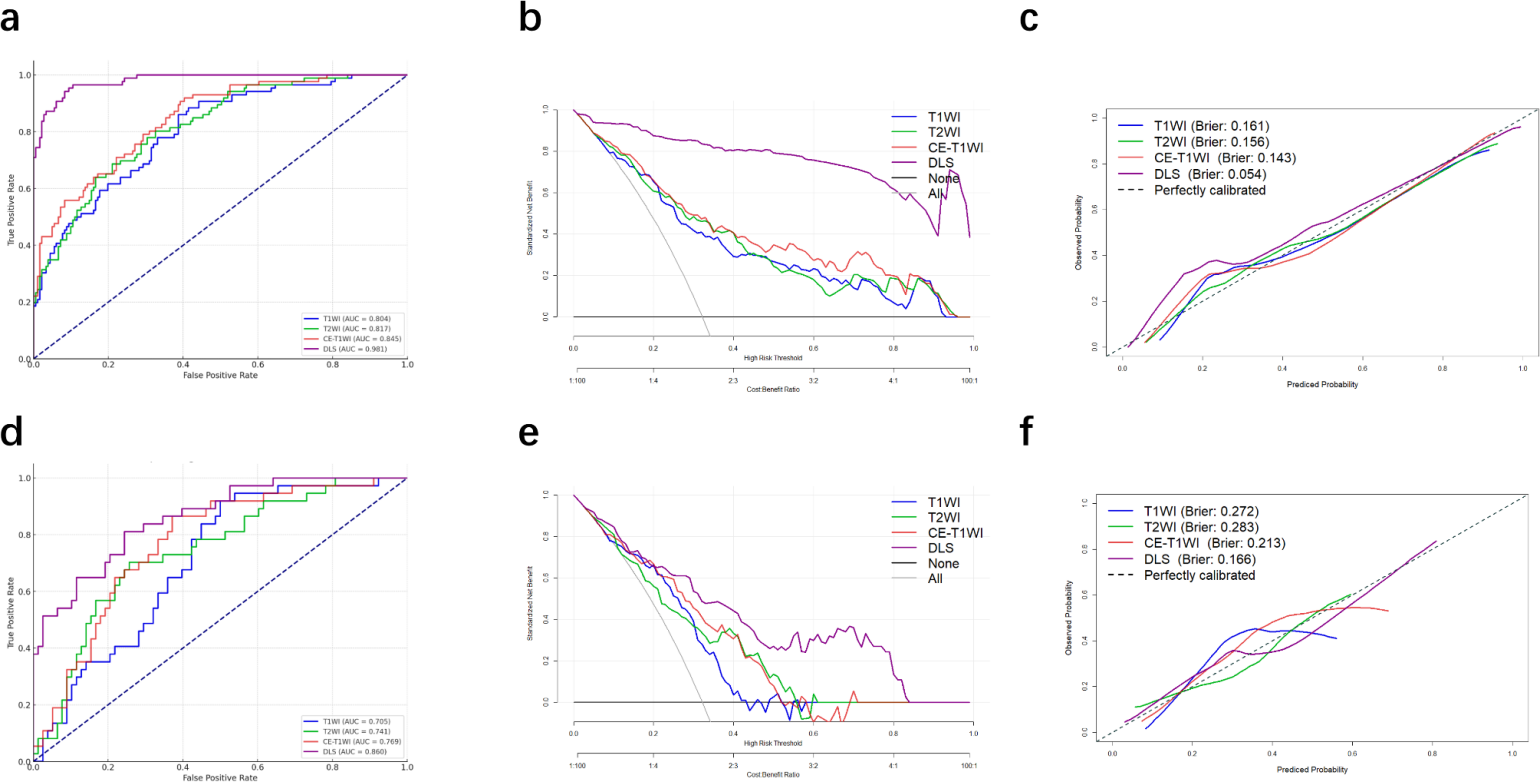
ROC curves, decision curve analysis, and calibration curves of different DL models in training cohort (**a**-**c**) and internal validation cohort (**d**-**f**)

Models were developed using T1WI, T2WI, and CE-T1WI sequences, both as individual and combined sequence models. To assess the impact of different sequences on model performance and identify the optimal predictive model, these models were evaluated in both the training and validation cohorts. The DLS achieved superior performance, with an AUC of 0.981, sensitivity of 0.826, and specificity of 0.978 (Table 2; Fig. 3a). In line with training results, DLS outperformed single-sequence models in the validation cohorts, achieving an AUC of 0.860, sensitivity of 0.811, and specificity of 0.756 (Table 2; Fig. 3d). The DeLong test confirmed statistically significant performance differences between the DLS and T1WI, T2WI, and CE-T1WI models in the internal validation cohort, with no significant difference compared to CE-T1WI (DLS vs. CE-T1WI, *P* = 0.149).

**Fig. 4:**
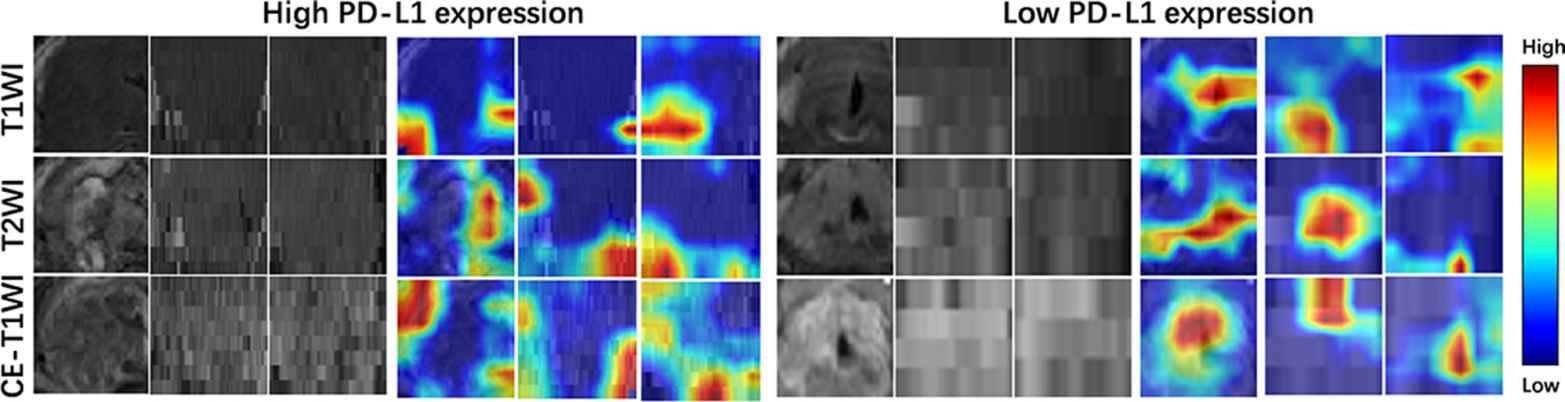
Grad-CAM heatmaps of head and neck squamous carcinoma patients with high PD-L1 expression and low PD-L1 expression in T1WI, T2WI, and CE-T1WI

To further evaluate the diagnostic accuracy of the DLS, we applied decision curve analysis (DCA), which showed that the DLS offered the highest net benefit (Fig. 3b and e). Furthermore, the classification accuracy of DLS, evidenced by superior Brier scores and calibration curves (Fig. 3c and f), outperformed other models in the internal validation cohort. Figure 4 illustrates the feature activation maps identified by our deep convolutional neural networks, specifically focusing on PD-L1.

**Table 2:** Diagnostic performance of models constructed by Resnet-50 on the training and validation cohorts

Group	Models	AUC (95%CI)	Accuracy	Sensitivity	Specificity	PPV	NPV
Training set	T1WI	0.804 (0.756–0.851)	0.764	0.419	0.928	0.735	0.771
	T2WI	0.817 (0.770–0.863)	0.760	0.419	0.923	0.720	0.770
	CE-T1WI	0.845 (0.802–0.889)	0.790	0.558	0.901	0.727	0.811
	DLS	0.981 (0.965–0.997)	0.929	0.826	0.978	0.947	0.922
Internal validation set	T1WI	0.705 (0.621–0.755)	0.626	0.865	0.513	0.457	0.889
	T2WI	0.741 (0.661–0.821)	0.565	0.865	0.423	0.416	0.868
	CE-T1WI	0.769 (0.692–0.846)	0.696	0.757	0.667	0.519	0.853
	combined	0.860 (0.797–0.924)	0.774	0.811	0.756	0.612	0.894
External validation set	T1WI	0.689 (0.611–0.768)	0.619	0.800	0.449	0.578	0.705
	T2WI	0.747 (0.674–0.821)	0.702	0.846	0.565	0.647	0.796
	CE-T1WI	0.734 (0.659–0.809)	0.642	0.600	0.681	0.639	0.644
	combined	0.803 (0.735–0.870)	0.754	0.708	0.797	0.767	0.743

AUC, area under the curve; CI, confidence interval; DL, deep learning; NPV, negative predictive value; PPV, positive predictive value

### External validation and survival analysis

**Fig. 5:**
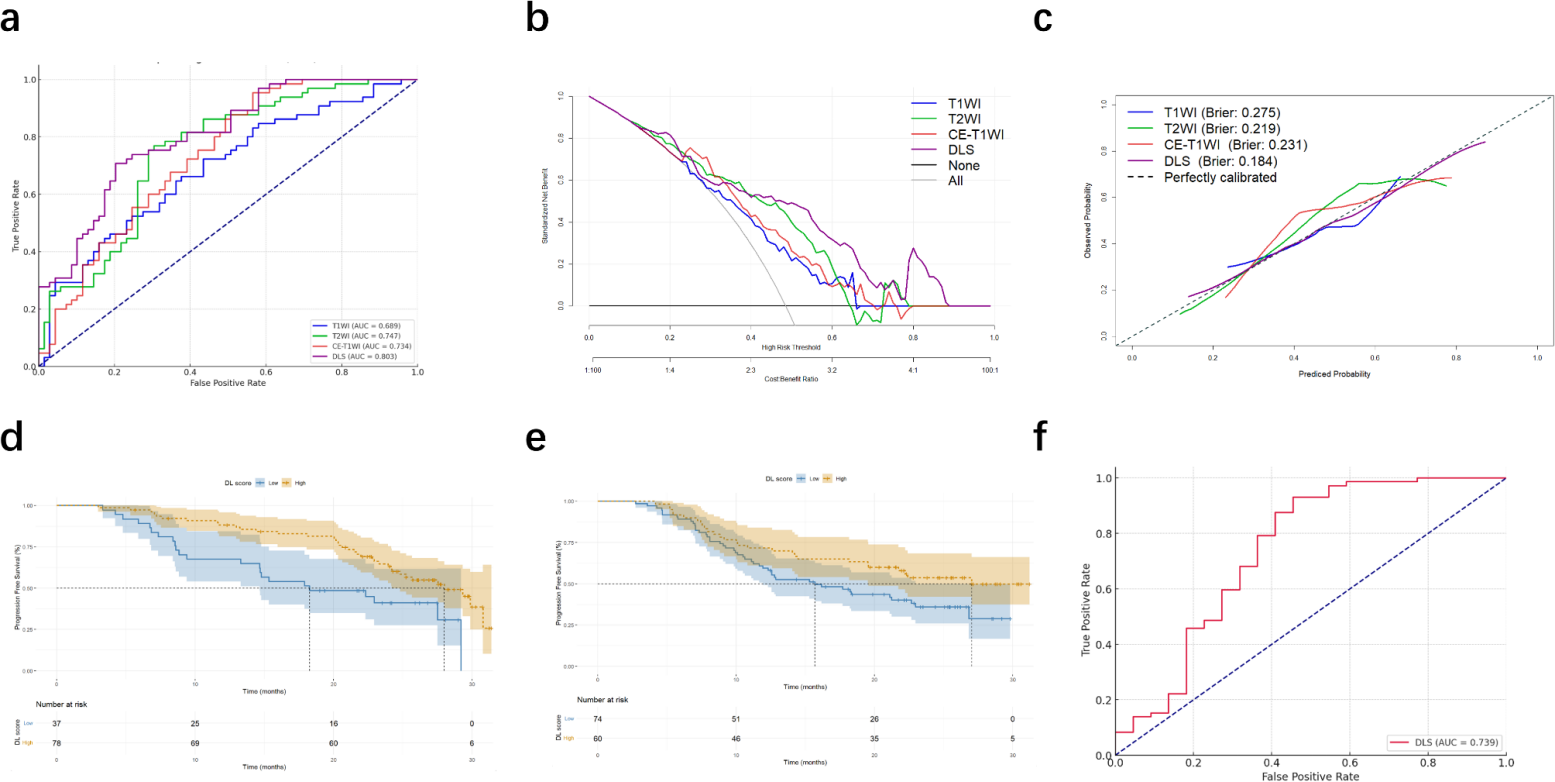
ROC curves, decision curve analysis, and calibration curves of different DL models in external validation cohort **(a-c)**; Kaplan-Meier survival curves for the DLS in the internal validation cohort **(d)**, and external validation cohort **(e)**. ROC curve of DLS for identifying DCB in the ICI-treated cohort **(f)**

To evaluate the robustness of the DLS, an external validation cohort of 197 patients was utilized. In the external validation cohort, the DLS showed stable performance, achieving an AUC of 0.803, with a sensitivity of 0.708 and a specificity of 0.797 (Fig. 5a). DCA indicated that the DLS provided the greatest net benefit (Fig. 5b). Furthermore, Brier scores and calibration curves clearly indicated that the classification accuracy of the DLS surpassed that of other single-sequence models in the external validation cohort (Fig. 5c).

To further assess the prognostic value of the deep learning model for HNSCC patients, Kaplan-Meier survival curves were generated for both the internal and external validation cohorts. As of July 2024, the median follow-up times were 22.80 months (IQR, 14.68–27.03 months) for the internal validation cohort and 18.28 months (IQR, 9.16–24.98 months) for the external validation cohort.

Using the Youden index from the training cohort, patients were categorized into high DLS and low DLS groups. Figure 5d and e show that the high DLS and low DLS groups displayed significant differences in both internal (hazard ratio 0.491; 95% CI, 0.270–0.892; *P* = 0.005) and external validation cohorts (hazard ratio 0.617; 95% CI, 0.391–0.973; *P* = 0.040). In the ICI-treated cohort, patients with DCB had significantly higher DLS scores, achieving an AUC of 0.739 for identifying DCB (95% CI: 0.639–0.824) (Fig. 5f).

## Discussion

According to NCCN guidelines, IHC assessment of PD-L1 expression serves as a decision-support tool for HNSCC patients considering checkpoint inhibitor therapy. In this study, the proposed DLS based on MRI to non-invasively assess PD-L1 status achieved high predictive accuracy, with area under the curve (AUC) values of 0.981 in the training cohort, 0.860 in the internal validation cohort, and 0.803 in the external validation cohort. Furthermore, higher DLS were significantly correlated with improved PFS, effectively stratifying patient prognosis. Additionally, the DLS identified patients likely to benefit from immunotherapy, achieving an AUC of 0.739 for predicting DCB in the immunotherapy cohort, which is crucial for developing personalized treatment strategies.

The administration of the PD-1/PD-L1 inhibitor pembrolizumab in HNSCC patients mainly depends on their PD-L1 expression levels. Studies have shown that patients with high PD-L1 expression (CPS ≥ 20) tend to respond better to pembrolizumab, while those with CPS below 20 are generally advised to receive pembrolizumab in combination with chemotherapy [7]. Artificial intelligence techniques, particularly radiomics and deep learning, are becoming increasingly prevalent in oncology research for medical imaging, including evaluations of head and neck tumors [24–26]. Although several studies have investigated non-invasive PD-L1 expression prediction using CT-based radiomics, their findings have been limited by small sample sizes and the radiation risks associated with CT imaging [14, 15]. In contrast, MRI poses no radiation risk and offers multiparametric imaging capabilities, thereby providing richer tumor information [27]. Previous research has demonstrated that multiparametric MRI radiomic features combined with deep learning methods outperform single-modality approaches [17, 28]. The American Journal of Roentgenology (AJR) recommends T1WI, T2WI, and CE-T1WI as standard imaging sequences for head and neck cancer, revealing tumors’ internal characteristics and blood supply [29]. Compared to radiomic models, deep learning minimizes the subjectivity and time involved in manual feature selection and utilizes a hierarchical structure of nonlinear features to more effectively model complex data patterns [30]. The DLS developed in this study demonstrated superior performance in predicting PD-L1 expression, with AUC values of 0.860 and 0.803 in the internal and external validation cohort. Furthermore, our analysis demonstrated that the DLS effectively stratifies patient prognosis, as patients with higher DLS scores experienced improved oncological outcomes and greater clinical benefits. These findings suggest that our DLS can guide personalized treatment decisions for HNSCC.

The ResNet network introduces residual learning, allowing for deeper network structures that efficiently retain and transmit gradient information during training, effectively capturing local image features [31]. Consequently, we employed the ResNet architecture to extract deep learning features from T1WI, T2WI, and CE-T1WI sequences, and utilized an attention mechanism to fuse these features. The attention mechanism simulates human selective attention, enhancing the model’s ability to capture essential information while reducing interference from irrelevant data [32]. Additionally, Grad-CAM heatmaps were employed for visual interpretation, clarifying the relationship between deep features and PD-L1 expression. This analysis revealed that certain salient features originate from tumor-adjacent areas, which is consistent with the findings of Austin et al. Austin et al.‘s research demonstrated that 83% of PD-L1 expression in HNSCC tumors is peripheral, with this staining pattern suggesting an induced response associated with inflammation, potentially the most sensitive to anti-PD-1 therapy [33]. These findings highlight the advantages of deep learning models in capturing features of tumors and their microenvironment, thereby enhancing clinical interpretability and addressing the opaque nature of deep learning.

However, the DLS faces several challenges in practical applications. Although previous studies have demonstrated the potential of automated segmentation techniques in HNSCC [34–36], the complexity of the head and neck region, coupled with the high heterogeneity in the size, shape, and location of HNSCC tumors, makes automated segmentation difficult when detecting small lesions or precisely delineating tumor boundaries—especially when the boundaries between the tumor and surrounding tissues are unclear [37]. Therefore, we opted for manual segmentation to achieve more accurate delineation of the tumor. However, manual segmentation is typically time-consuming, and discrepancies between experts may impact the accuracy of the model. Consequently, further development of an end-to-end model is crucial to promote its broader clinical application. Furthermore, while the DLS demonstrated high diagnostic efficacy in predicting PD-L1 expression, its AUC for predicting DCB is relatively modest. The possible reason for this is the inclusion of both PD-L1 high and low expression patients in the ICI treatment cohort of this study. Several studies have shown that a small subset of PD-L1-negative or low-expression patients can also benefit from ICI treatment [38, 39]. Therefore, future research should focus on identifying those patients who are likely to benefit from immunotherapy, particularly PD-L1-negative or low-expression patients. Lastly, while DLS holds potential in predicting PD-L1 expression in HNSCC patients, especially in assessing the efficacy of ICI treatment, IHC testing remains the primary method for guiding clinical decision-making. Therefore, future studies should explore the integration of DLS with IHC testing to provide a more comprehensive evaluation tool, helping to early identify patients who may benefit from immunotherapy, thereby enabling more precise and personalized treatment decisions.

Several limitations were identified in this study. First, this study retrospectively included patients from two centers, resulting in a limited sample size and potential selection bias. Therefore, future validation of the model should be performed using multi-center data and prospective cohorts. Secondly, only IHC, as recommended by the NCCN Clinical Practice Guidelines, was utilized to assess PD-L1 levels, while other methods such as immunofluorescence and flow cytometry were not employed. Future research should compare these detection methods, although all require biopsy. Thirdly, MRI was used in this study to assess PD-L1 expression and the efficacy of immunotherapy; however, this method may not be widely applicable in all clinical settings. Although MRI has the advantages of no radiation exposure and multiparametric imaging, CT is more commonly used in clinical practice and is widely employed for the diagnosis and monitoring of HNSCC. Therefore, future studies should further explore deep learning models based on CT to assess PD-L1 expression and the efficacy of immunotherapy in HNSCC, which could improve its applicability in clinical settings.

## Conclusions

In conclusion, DLS demonstrated satisfactory predictive performance in external cohorts and may serve as a prognostic biomarker to guide immunotherapy. These findings suggest that DLS could be used to identify patients likely to benefit from immunotherapy prior to.

## Electronic supplementary material

Below is the link to the electronic supplementary material.

Supplementary Material 1: Methods S1 Inclusion and exclusion criteria. Table S1 MR scanning parameters. Table S2 Clinical Characteristics of ICI cohort.

## Data Availability

No datasets were generated or analysed during the current study.

## Abbreviations

NCCN: National Comprehensive Cancer Network
HNSCC: Head and neck squamous cell carcinoma
DLS: Deep learning score
LR: Logistic regression
DCB: Durable clinical benefit
ICI: Immune checkpoint inhibitors
ROC: Receiver operating characteristic
MRI: Magnetic resonance imaging
PFS: Progression-free survival
ROI: Region of interest
CPS: Combined positivity score
ESMO: European Society for Medical Oncology
ASCO: American Society of Clinical Oncology
IHC: Immunohistochemistry
T1WI: T1-weighted imaging
T2WI: T2-weighted imaging
CE-T1WI: Contrast-enhanced T1-weighted imaging
AUC: Area under the curve
Grad-CAM: Gradient-weighted Class Activation Mapping
PCA: Principal Component Analysis
MLP: Multilayer perceptron
CNN: Convolutional Neural Networks
SGD: Stochastic Gradient Descent
CI: Confidence interval
NPV: Negative predictive value
PPV: Positive predictive value
DCA: Decision curve analysis
AJR: American Journal of Roentgenology
