# Supplementary material for: Development and validation of MRI-derived deep learning score for non-invasive prediction of PD-L1 expression and prognostic stratification in head and neck squamous cell carcinoma
Source: Cancer Imaging. 2025 Feb 16;25:14. doi: 10.1186/s40644-025-00837-5 (PMC11831796; doi:10.1186/s40644-025-00837-5)
Supplement: Supplementary file 1 — Supplementary Material 1: Methods S1 Inclusion and exclusion criteria. Table S1 MR scanning parameters. Table S2 Clinical Characteristics of ICI cohort. [file 40644_2025_837_MOESM1_ESM.docx]

Methods S1

Inclusion and exclusion criteria

For PD-L1 prediction, patients with informed consent were accrued from the Beijing Tongren Hospital, Beijing, China, between 6 2021 and 6 2024 with following inclusion criteria were included: 1) histologically confirmed primary HNSCC; 2) pathological examination of PD-L1 status before any treatment; 3) MRI scans obtained within one month before biopsy for immunohistochemistry (IHC) and no treatments was performed during this interval; 5) baseline clinical characteristics (including age, sex, TN stage, histology, and smoking history) were available. Based on these inclusion criteria, 382 patients were identified and subsequently assigned to a training cohort (Tongren training, N = 267) and an independent test cohort (Tongren-test, N = 115). Using the same inclusion criteria, 134 HNSCC patients were accrued from The Affiliated Huaian No. 1 People’s Hospital of Nanjing Medical University, Huaian, Jiangsu and were used as external independent test cohort for prediction of PD-L1 expression (Huaian-PD-L1 cohort).

For the distinct cohorts to predict patient response and outcomes, 94 patients were identified with histologically confirmed HNSCC who were treated with immunotherapy (anti-PD-L1 or anti-PD-1) between 6 2021 and 3 2024 at Beijing Tongren Hospital, Beijing, China, using the following criteria: 1) MRI scans obtained within one month before biopsy (Bx) for immunohistochemistry (IHC) and no treatments was performed during this interval; 3) follow-up time was greater than 6 months; and 4) no immune-related severe adverse events (Grade according to Common Terminology Criteria for Adverse Events (CTCAE)>=3S1) were observed or reported during treatment; 5) baseline clinical characteristics (including age, sex,TN stage, histology, and smoking history) were available.

| Table S1. MR scanning parameters | | | | | | | | | |
| --- | --- | --- | --- | --- | --- | --- | --- | --- | --- |
| Group | MR scanner and sequence | TR (ms) | TE (ms) | Matrix | FOV (cm2) | Slice thickness (mm) | Number of slices | Slice gap (mm) | NEX |
| Center1 | PHILIPS Ingenia 3.0T |  |  |  |  |  |  |  |  |
|  | T1WI | 600-700 | 6-7 | 352×228 | 21×19 | 4 | 20 | 0.4 | 2 |
|  | T2WI | 2500-3000 | 80-90 | 352×249 | 21×19 | 4 | 20 | 0.4 | 2 |
|  | CE-T1WI | 600-700 | 7-8 | 264×201 | 21×19 | 4 | 20 | 0.4 | 2 |
|  | GE Signa HDxt 3.0T |  |  |  |  |  |  |  |  |
|  | T1WI | 400-500 | 10 | 320×256 | 22×22 | 4-5 | 20 | 0.5 | 2 |
|  | T2WI | 3500-4000 | 90 | 512×256 | 22×22 | 4-5 | 20 | 0.5 | 2 |
|  | CE-T1WI | 400-500 | 10 | 320×224 | 22×22 | 4_5 | 20 | 0.5 | 2 |
|  | GE Discovery MR750 3.0T |  |  |  |  |  |  |  |  |
|  | T1WI | 560-590 | 10 | 288×224 | 22×22 | 4-4.5 | 24 | 0.3-0.4 | 2 |
|  | T2WI | 3975-4720 | 85-90 | 320×224 | 22×22 | 4-4.5 | 24 | 0.3-0.4 | 2 |
|  | CE-T1WI | 560-590 | 7-8 | 288×224 | 22×22 | 4-4.5 | 24 | 0.3-0.4 | 2 |
|  | SIEMENS Prisma 3.0T |  |  |  |  |  |  |  |  |
|  | T1WI | 600-700 | 9-10 | 320×224 | 21×19 | 4 | 24 | 0.4 | 2 |
|  | T2WI | 6000-7000 | 90-110 | 448×314 | 21×19 | 4 | 24 | 0.4 | 2 |
|  | CE-T1WI | 600-700 | 12 | 320×224 | 21×19 | 4 | 24 | 0.4 | 2 |
| Center2 | GE Discovery MR750 3.0T |  |  |  |  |  |  |  |  |
|  | T1WI | 600-800 | 8-10 | 288×192 | 22×22 | 6 | 24 | 1 | 2 |
|  | T2WI | 2500-3550 | 90-100 | 288×193 | 22×22 | 6 | 24 | 1 | 2 |
|  | CE-T1WI | 600-800 | 8-10 | 288×194 | 22×22 | 6 | 24 | 1 | 2 |
|  | PHILIPS Ingenia 3.0T |  |  |  |  |  |  |  |  |
|  | T1WI | 400-500 | 6-8 | 308×230 | 21×19 | 6 | 24 | 7 | 2 |
|  | T2WI | 2000-3550 | 85-100 | 288×208 | 21×19 | 6 | 24 | 7 | 2 |
|  | CE-T1WI | 500-600 | 14 | 272×214 | 21×19 | 4-4.4 | 24 | 0.3-0.4 | 2 |
| Center3 | GE Discovery MR750 3.0T |  |  |  |  |  |  |  |  |
|  | T1WI | 400-500 | 9_10 | 288×224 | 22×22 | 4 | 20 | 1 | 2 |
|  | T2WI | 2500-3000 | 70-80 | 288×192 | 22×22 | 4 | 24 | 1 | 2 |
|  | CE-T1WI | 500-600 | 9-10 | 288×192 | 22×22 | 4 | 24 | 1 | 2 |
|  | PHILIPS Ingenia 3.0T |  |  |  |  |  |  |  |  |
|  | T1WI | 550-700 | 9-10 | 316×222 | 21×19 | 4 | 20 | 0.8 | 2 |
|  | T2WI | 2000-3000 | 80-90 | 340×235 | 21×19 | 4 | 20 | 0.8 | 2 |
|  | CE-T1WI | 550-700 | 15-25 | 316×215 | 21×19 | 4 | 20 | 0.4 | 2 |
|  | SIEMENS Skyra 3.0T |  |  |  |  |  |  |  |  |
|  | T1WI | 200-300 | 2-7 | 320×240 | 21×19 | 4 | 20 | 1.2 | 2 |
|  | T2WI | 3500-4500 | 90-120 | 320×218 | 21×19 | 4 | 20 | 1.2 | 2 |
|  | CE-T1WI | 650-850 | 9-10 | 320×224 | 21×19 | 4 | 20 | 1.2 | 2 |
| Center4 | GE Signa HDxt 3.0T |  |  |  |  |  |  |  |  |
|  | T1WI | 300-500 | 10-20 | 320×256 | 22×22 | 3 | 24 | 0.3 | 2 |
|  | T2WI | 4000-6600 | 68-80 | 320×256 | 22×22 | 3 | 24 | 0.3 | 2 |
|  | CE-T1WI | 300-500 |  | 320×256 | 22×22 | 3 | 24 | 0.3 | 2 |
|  | SIEMENS Prisma 3.0T |  |  |  |  |  |  |  |  |
|  | T1WI | 600-700 | 9-10 | 320×256 | 22×22 | 3 | 24 | 0.3 | 2 |
|  | T2WI | 6000-7000 | 90-110 | 320×256 | 22×22 | 3 | 24 | 0.3 | 2 |
|  | CE-T1WI | 600-700 | 12 | 320×256 | 22×22 | 3 | 24 | 0.3 | 2 |
| TR, repetition time; TE, echo time; FOV, Field of view; NEX, number of excitations; FSE, fast spin echo; TSE, turbo spin echo. | | | | | | | | |  |

|  |  | Table S2. Clinical Characteristics of ICI cohort | | | | | | |
| --- | --- | --- | --- | --- | --- | --- | --- | --- |
| Clinical Characteristics | | |  | Non-DCB | DCB |  | P value |  |
| Age (mean ± SD, years) | | |  | 59.68+9.81 | 58.53+9.31 |  | 0.737 |  |
| Histological differentiation | | |  |  |  |  | 0.302 |  |
|  | | | Well | 2 (9.09%) | 5 (6.94%) |  |  |  |
|  | | | Moderately | 6 (27.27%) | 33 (45.83%) |  |  |  |
|  | | | Poorly | 14 (63.64%) | 34 (47.22%) |  |  |  |
| T stage | | |  |  |  |  | 0.824 |  |
|  | | | 2 | 3 (13.64%) | 14 (19.44%) |  |  |  |
|  | | | 3 | 11 (50.00%) | 34 (47.22%) |  |  |  |
|  | | | 4 | 8 (36.36%) | 24 (33.33%) |  |  |  |
| N stage | | |  |  |  |  | 0.375 |  |
|  | | | 0 | 0 | 5 (6.94%) |  |  |  |
|  | | | 1 | 11 (50.00%) | 36 (50.00%) |  |  |  |
|  | | | 2 | 9 (40.91%) | 29 (40.28%) |  |  |  |
|  | | | 3 | 2 (9.09%) | 2 (2.78%) |  |  |  |
| Smoking | | |  |  |  |  | 0.802 |  |
|  | | | No | 8 (36.36%) | 22 (30.56%) |  |  |  |
|  | | | Yes | 14 (63.64%) | 50 (69.44%) |  |  |  |
| Drink | | |  |  |  |  | 0.434 |  |
|  | | | No | 12 (54.55%) | 48 (66.67%) |  |  |  |
|  | | | Yes | 10 (45.45%) | 24 (33.33%) |  |  |  |
| Sex | | |  |  |  |  | 0.336 |  |
|  | | | Male | 16 (72.73%) | 61 (84.72%) |  |  |  |
|  | | | Female | 6 (27.27%) | 11 (15.28%) |  |  |  |
| P value < 0.05 is considered as a significant difference.SD, standard deviation. | | | | | | | | |
|  |  |  | | | | | | |
